# Novel selectively amplified DNA sequences in the germline genome of the Japanese hagfish, *Eptatretus burgeri*

**DOI:** 10.1038/s41598-022-26007-2

**Published:** 2022-12-09

**Authors:** Kohei Nagao, Tomoko Otsuzumi, Hitomi Chinone, Takashi Sasaki, Junko Yoshimoto, Makiko Matsuda, Souichirou Kubota, Yuji Goto

**Affiliations:** Department of Biology, Faculty of Science, Toho University, Miyama 2-2-1, Funabashi, Chiba 274-8510 Japan

**Keywords:** Cytogenetics, Molecular evolution, Chromosomes, Genome evolution, Mobile elements

## Abstract

In the Japanese hagfish *Eptatretus burgeri*, 16 chromosomes (eliminated [E]-chromosomes) have been lost in somatic cells (2n = 36), which is equivalent to approx. 21% of the genomic DNA in germ cells (2n = 52). At least seven of the 12 eliminated repetitive DNA families isolated in eight hagfish species were selectively amplified in the germline genome of this species. One of them, EEEb1 (eliminated element of *E.* *burgeri* 1) is exclusively localized on all E-chromosomes. Herein, we identified four novel eliminated repetitive DNA families (named EEEb3–6) through PCR amplification and suppressive subtractive hybridization (SSH) combined with Southern-blot hybridization. EEEb3 was mosaic for 5S rDNA and SINE elements. EEEb4 was GC-rich repeats and has one pair of direct and inverted repeats, whereas EEEb5 and EEEb6 were AT-rich repeats with one pair and two pairs of sub-repeats, respectively. Interestingly, all repeat classes except EEEb3 were transcribed in the testes, although no open reading frames (ORF) were identified. We conducted fluorescence in situ hybridization (FISH) to examine the chromosomal localizations of EEEb3–6 and EEEb2, which was previously isolated from the germline genome of *E. burgeri.* All sequences were only found on all EEEb1-positive E-chromosomes. Copy number estimation of the repeated elements by slot-blot hybridization revealed that (*i*) the EEEb1–6 family members occupied 39.9% of the total eliminated DNA, and (*ii*) a small number of repeats were retained in somatic cells, suggesting that there is incomplete elimination of the repeated elements. These results provide new insights into the mechanisms involved in the chromosome elimination and the evolution of E-chromosomes.

## Introduction

In general, all cells within multicellular organisms are genetically identical throughout their development and differentiation. In some organisms however, the number of chromosomes and/or the amount of DNA differs significantly between germ and somatic cells. This unusual phenomenon was first discovered by Boveri in the nematode *Parascaris equorum*^1^. In the early embryogenesis of *P. equorum*, presumptive germ cells have one pair of large chromosomes (2n = 2), but these chromosomes are broken into numerous smaller chromosomes and some larger chromosomes during early development, and subsequently, large portions of the chromosomes are excluded from presumptive somatic cells at 2nd to 5th cleavage division. The elimination of specific chromosomal fragments or entire chromosomes from germ cells called chromosomal elimination and chromatin diminution respectively occurs in several species of nematodes, insects, crustaceans, and vertebrates, and is collectively referred to as programmed genome rearrangement (PGR)^2^. While the eliminated chromosomes (E-chromosomes) are typically composed of constitutive heterochromatin and highly repetitive DNA sequences, there are protein coding genes that could be involved in early stages of embryogenesis or gonadogenesis, as reported in songbird and sea lamprey^3–6^.

Chromosome elimination was first described in vertebrates by Kohno et al*.* in the Japanese hagfish, *Eptatretus burgeri*^7^. This phenomenon has now been observed in seven other hagfish species: *E.* *stoutii*, *E.* *okinoseanus*, *E.* *cirrhatus*, *Paramyxine atami*, *P.* *sheni*, *Myxine glutinosa*, and *M.* *garmani*^8^. In these species, two to 62 chromosomes are eliminated from presumptive somatic cells, which leads to the elimination of 20.9–74.5% of the germline genomic DNA. Subsequent molecular genetic analyses revealed that 12 highly repetitive DNA families are restrictively highly abundant in the germline genome and are eliminated from presumptive somatic cells^9–13^. Four of these families, i.e., EEEo1 (eliminated element of *E.* *okinoseanus* 1), EEEo2, EEPa1, and EEEb2 have been conserved in the germline genomes of the examined hagfish species over considerable evolutionary periods^9–13^.

In other agnathans, PGR has been reported from eight lamprey species, *Petromyzon marinus*, *Lampetra aepyptera*, *L. appendix*, *L. morii*, *Ichthyomyzon fossor*, *I. gagei*, *I. greeleyi* and *Entosphenus tridentatus*^14–17^. Although the number of eliminated chromosomes has been unclear owing to large number of small-dot shaped chromosomes, quantification of the nucleic DNA in germline and somatic cells clearly revealed the elimination of approximately 15% and 20% of *L. morii* and *P. marinus* germline genome from presumptive somatic cells, respectively^14,15^. Molecular genetic analyses revealed that nine repetitive DNA families are germline-specifically abundant in *P. marinus*. One of these DNA families, *Germ*1 contains a truncated 28S rDNA that was identified in the eliminated chromosomal regions from six lamprey species, implying that PGR may be a mechanism by which dysfunctional rDNAs are eliminated^17^.

In *E. burgeri*, the spermatogonial karyotype consists of 52 dot-shaped chromosomes including 36 C-band-negative and 16 C-band-positive chromosomes, whereas the somatic karyotype consists of 36 C-band-negative chromosomes, suggesting the exclusive elimination of C-band-positive chromosomes^7^. A DNA quantification of the germline and somatic genome demonstrated that the 16 E-chromosomes are equivalent to ~ 20.9% of the total nuclear DNA in the germline cells. Approximately 90% of the eliminated DNA consists of seven of the 12 known eliminated DNA families (EEEb1, EEEb2, EEEo1, EEEo2, EEPa1, EEPs1, and EEPs4), and Southern- and slot-blotting assays revealed that these families are characterized as a germline-restricted sequence of *E.* *burgeri*^9–11,13^. One of the eliminated DNA families, i.e., EEEb1, is exclusively localized on all E-chromosomes^11^.

Despite vigorous efforts to find eliminated genomic sequences of *E.* *burgeri*, potentially functional genes from the eliminated genome have been never identified. In most eukaryotes, repetitive DNA sequences are widely distributed in the genome and located mainly in the constitutive heterochromatin, but their function(s) are poorly understood. One of the most well-known examples of functional repetitive sequences is rRNA gene (rDNA). The rDNAs are arranged into two distinct clusters: the major (18S, 5.8S, and 28S rDNAs) cluster and the minor (5S rDNA) cluster. In some fish species, 5S rDNA is differentiated into two types (germline and somatic types) and differentially expressed between germline and somatic cells^18–20^.

Studies of nematodes revealed that the eliminated genome contains at least 685 predicted genes, and one of them (named *rpS19G*) encodes a homologue to the 40S ribosomal protein S19 of eukaryotes^21,22^. Similar results were reported in sea lamprey and zebra finch, in which protein-coding genes are also eliminated during early embryogenesis^,^^3,6,23^. Hence, these studies suggest that the biological functions of PGR include its’ ability to remove functional protein-coding genes and provide a mechanism by which irreversibly gene silencing can occur to avoid potentially misexpression in somatic lineage.

In the present work, we attempted to identify eliminated genes of *E.* *burgeri* by two approaches. We first investigated whether two types of 5S rDNA sequences exist in the *E.* *burgeri* genome by conducting molecular cloning via PCR amplification. We then investigated whether genes that are expressed specifically in the germline are present in the eliminated *E.* *burgeri* genome. For this purpose, we applied a suppressive subtractive hybridization (SSH) technique that is used for the selective amplification of differentially expressed sequences^24^. We also used molecular genetic techniques to analyze the genomic organization, chromosomal localization, copy number, and sequence characteristics of the eliminated DNA sequences detected in this hagfish species.

## Materials and methods

### Ethical approval

All animal experiments in this study were approved (Protocol#21-52-446) and conducted following the guidelines established by the Institutional Animal Care and Use Committee of Toho University. The study was carried out in compliance with the ARRIVE guidelines.

### Animals

Japanese hagfish *Eptatretus burgeri* were collected from Misaki Bay and Katase Bay in Kanagawa, Japan. The fish were injected intraperitoneally with colchicine (0.375 mg/kg bodyweight) 2 h before sacrifice to enrich mitotic cells. All animals were euthanized by a high dose of ethyl m-aminobenzoate methanesulfonate (MS-222) (Nacalai Tesque, Kyoto, Japan).

### Molecular cloning of rDNA-related sequences

PCR amplification of rDNA was performed on genomic DNA extracted from germline and somatic tissues including testes, peripheral blood cells, and liver by a standard protocol using proteinase K, phenol/chloroform extraction, and ethanol precipitation as previously described^9^.

The amplification of 5S rDNA was performed using TaKaRa Taq™ polymerase (TaKaRa Bio, Shiga, Japan) using 500 ng of each genomic DNA and the pair of primers 5Sr-1a and 5Sr-1b (Table 1), designed from the most conserved region in the reported 5S rDNA sequences^7,19,25–29^ according to the manufacturer's instructions. The amplification conditions were 30 cycles of 94 °C for 30 s, 60 °C for 2 min, and 73 °C for 30 s, and a final extension at 72 °C for 5 min. The PCR products were separated on 1% agarose gels and purified and ligated into the *Eco* RI site of plasmid pUC119 or pT7Blue Vector (Novagen, Darmstadt, Germany). After transformation into *Escherichia coli* strain JM109 and subsequent blue/white selection on ampicillin plates, plasmid DNAs from positive clones were prepared and the inserted DNA was sequenced as described^30^.

**Table 1:** Oligonucleotides used in this study.

Oligonucleotide	Oligonucleotide sequence (5′–3′)	Method
5Sr-1a	GAGACTGCCTGGGAATACC	Primer for PCR amplification of rDNA-related sequence
5Sr-1b	GTCTCCCATCAAGTACTAACC	
TrA-2a	CAACATGATTGGTCCA	Primer for PCR amplification of genomic SSH A sequence
TrA-2b	GGGTGATTTACCTCGA	
TrB-1a	GGCTCATAGAAGGAAGAAGC	Primer for PCR amplification of genomic SSH B sequence
TrB-1b	CAACATCGCCACCTACATG	
EEEb6-1a	CTCAGTAAKAAACRTCG	Primer for PCR amplification of genomic SSH C sequence
EEEb6-1b	GTTAAACCACGCCCAC	
Nested PCR Primer 1	TCGAGCGGCCGCCCGGGCAGGT	Primer for direct colony PCR
Nested PCR Primer 2R	AGCGTGGTCGCGGCCGAGGT	
M13-F	GTAAAACGACGGCCAGT	
M13-R	CAGGAAACAGCTATGAC	
EEEb3-f	AAATGCTGAGCATCGCG	Insert PCR for Southern-blot hybridization
EEEb3-r	GGCCAGAGAACTTGTG	
EEEb1-probe	GTTTCATGGTTATGGACCGTCGTTCCGCGTCGGCGTGTGA	Probe for slot-blot hybridization
EEEb2-probe	AAAGTTTGAGTGGTGTAAGAAGTTATAATGTTTTTTTTGAGGTGAGATGTTTATTTT	
EEEo1-probe	TATCTCAAAAAGTTGTKTAYGGNTTCGGAYGAAACTTGGTGGACACGTTGG	
EEEo2-probe	TGGAAGTGATTTTTTCTCCTTTTTTGTGGTCAGAAGTGGTTATTTTTCATTGTTTTT	
EEPa1-probe	AAAGTGAAAAGTGCCCCCTGGTGGATGATGGAAGGA	
EEPs1-probe	CGGAACTCGTCGTGCGTTTTGCACATGCGCGAACGACCCC	
EEEb4-probe	CCAGCGCGATCACGTGCCGCCGAGTAGAAGATGTGCACCT CGAGGTAAATCACCCAACATGATTGGT	
EEEb5-probe	CTCATAGAAGGAAGAAGCATCACATGATGTTATATGGACATGTAGGTGGCGATGTTGG	
EEEb6-probe	GGTGTGGGCGTGGTTTAACTCAGTAAGAAACGTCGAGAATGTAATTTTATAAATCG	
EEEb3-f	AAATGCTGAGCATCGCG	Primer for recloning of EEEb3
EEEb3-1b	GTGCCATGTTTGGTGGTATAA	

### Construction of the SSH cDNA library

Suppressive subtractive hybridization (SSH) is a PCR-based technique and is used to selectively amplify differentially expressed sequences. The principle of SSH has been described^24^. Briefly, cDNA is first synthesized from two different cell populations to be compared. The cDNA population containing specific transcripts to be extracted is called the ‘tester cDNA,’ and the reference cDNA population is called the ‘driver cDNA.’

The tester cDNA was subdivided into two samples and individually ligated to adaptor-A and adaptor-B at their 5’-ends, respectively. In the present study, the adaptor-A and adaptor-B ligated cDNAs were separately hybridized with an excess of driver cDNA. The two hybridization samples were mixed and hybridized with an excess of driver cDNA, and then the 3′-ends of single-stranded adaptors were filled in to create the primer annealing sites for PCR amplification. Exponential amplification can occur when two different adaptor sequences are present on their ends.

We isolated poly(A) RNAs of mature testis, peripheral blood, and liver of the adult male hagfish by using the Micro-FastTrack 2.0 mRNA Isolation Kit (Invitrogen, Carlsbad, CA), and then performed the cDNA synthesis and SSH library construction by using the PCR-Select cDNA Subtraction Kit (Clontech, Mountain View, CA) with TaKaRa Taq™ according to the recommended protocols. With the use of cDNA from testes as the tester cDNA and cDNA from somatic cells (peripheral blood + liver) as the driver cDNA, the successfully subtracted cDNA fragments were inserted into the pCR2.1-TOPO-TA vector (Thermo Fisher Scientific, Waltham, MA). After transformation into TOP10 chemically competent cells (Thermo Fisher Scientific) and blue/white selection on the kanamycin plates, the size of the inserted DNA in each clone was certified by a direct colony PCR with nested PCR primer 1 and nested PCR primer 2R (Table 1). Plasmid DNA from the positive clones was isolated as described^30^.

### Screening of the SSH cDNA library

The germline specificity of the cloned sequence was confirmed by two rounds of dot-blot hybridization analyses. In the first round, the germline and somatic cDNA probes digested with *Rsa* I (Fujifilm Wako, Tokyo) were labeled with alkaline-phosphatase and hybridized with 50 µg of the denaturated plasmid DNAs blotted by a Bio-Dot Microfiltration Apparatus (PerkinElmer Life Sciences, Boston, MA). Labeling and detection of the probes were achieved with an AlkPhos Direct Labeling and Detection System with CDP-*Star* (GE Healthcare, Buckinghamshire, UK) according to the manufacturer’s instructions. The chemiluminescence signal was detected by X-ray film.

In the second round, the positive cDNA clones of the 1st round were used as probes and hybridized to 2.4 µg of somatic DNA and 3.0 µg of germline DNA, respectively. Dot-blot hybridization was performed under the same conditions. The nucleotide sequences of the inserts were determined by dye terminator sequencing, using the DTCS Quick Start Kit (Beckman Coulter, Indianapolis, IN) and the Genetic Analyzer CEQ8000 (Beckman Coulter) according to the manufacturer’s recommended protocol.

### Molecular cloning of the candidates isolated by SSH analysis from genomic DNA

Three germline-specific candidates from the SSH analysis, i.e., SSH A, B, and C, were amplified using TaKaRa Taq™ polymerase with 5 ng of germline DNA and each primer pair designed from the consensus sequences of each cDNA clone (Table 1). SSH A was amplified under the following conditions: an initial denaturation at 95 °C for 2 min, 30 cycles of 94 °C for 20 s, 50.1 °C for 20 s, and 72 °C for 30 s, and 72 °C for 3 min. For SSH B, the PCR conditions were changed as follows: 30 cycles of 94 °C for 20 s, 64 °C for 20 s, and 72 °C for 30 s. The PCR for SSH C was also altered as follows: 30 cycles of 94 °C for 20 s, 50.4 °C for 20 s, and 72 °C for 15 s. After the extraction and purification of the PCR products from agarose gels using a QIAquick Gel Extraction Kit (Qiagen, Hilden, Germany), the PCR products of SSH A, B, and C were ligated into pCR2.1-TOPO-TA vector and transformed into TOP10 chemically competent cells. The insertion and nucleotide sequence of positive clones were verified by colony PCR using the universal primers M13-F and -R (Table 1) and dye terminator sequencing as described above.

### Sequence analysis of identified sequences

All nucleotide sequences were aligned by the software program Genetyx-Mac ver. 21.0.0, which was manually modified if necessary. Gap sites were not included in the calculation of intraspecific sequence diversity. A homology search was accomplished by using the DNA databases blastn-NCBI (https://blast.ncbi.nlm.nih.gov/Blast.cgi) and Repbase-GIRI (https://www.girinst.org/repbase/).

### Southern-blot hybridization

Southern-blot hybridization was performed using 3 μg of germline and somatic genomic DNA digested with seven restriction endonucleases under the conditions outlined by the suppliers: *Msp* I, *Rsa* I, *Sau*96 I, *Xho* I (Nippon Gene, Tokyo), *Dde* I (Toyobo, Osaka, Japan), *Nsp* I (New England BioLabs, Ipswich, MA), and *Mbo* II (TaKaRa Bio). The digested DNAs were separated on a 1.2% agarose gel (SSH A and B) or a 1.5% agarose gel (SSH C) and transferred to a GeneScreen Plus hybridization transfer membrane (PerkinElmer Life Sciences) by the downward alkaline capillary transfer method^31^.

Probes were prepared for the region of Eb-G-5S homologous to the SINE2 family. The probes were prepared from cloned 5S rDNA (see “Results”), amplified from the plasmid pT7-EbG-3097 by insert PCR using TaKaRa Taq™ polymerase under the following conditions: 95 °C for 2 min, 30 cycles of 94 °C for 30 s, 55 °C for 30 s, and 72 °C for 30 s, and 72 °C for 1 min. Probes for SSH A, SSH B, and SSH C were prepared by the digestion of plasmids TrA-1-8, TrB-1-17, and T2-49 with *Eco*RI (TaKaRa), respectively. Labeling of the probes, hybridization, washing and detection were carried out as described in the text above regarding dot-blot hybridization. The sequence data used in this experiment have been deposited in GenBank (LC669413 and LC669415–LC669417).

### FISH

Fluorescence in situ hybridization (FISH) was performed using chromosome slides from the hagfish testes and gills prepared as described by Goto et al.^32^ with slight modification of the duration of hypotonic treatment from 10 to 30 min. These slides were treated with 100 μg/mL RNase A (type I-AS; Merck, Darmstadt, Germany) in 1 × SSC (standard saline citrate) for 30 min at 37 °C, followed by dehydration and drying through 70% and 100% ethanol series. The plasmid DNAs, pT7-EbG-3097, TrA-1-8, TrB-1-17, and T2-49 were labeled with biotin-16-dUTP (deoxyuridine triphosphate) (Promo Kine, Heidelberg, Germany), and plasmid DNA harboring EEEb1 was labeled with digoxigenin-11-dUTP (Enzo Life Sciences, New York, NY) by nick-translation as described by Green and Sambrook^33^.

After ethanol precipitation with 25 μg of yeast tRNA (Invitrogen), labeled probe DNA was thoroughly resuspended in 20 μL of formamide, and denaturation was then performed at 75 °C for 10 min. The denaturation of chromosomal DNA, hybridization, washing, and detection were performed as described^9^ with slight modifications. Chromosomal DNA was denatured with 70% formamide/2 × SSC at 70 °C for 2 min and then immediately dipped in ice-cold 70% and 100% ethanol for 5 min, respectively. Approximately 500 ng of probe DNA was applied per slide in 20 µL of hybridization mixture (2 × SSC, 2 mg/mL bovine serum albumin [BSA], 10% dextran sulfate). After overnight hybridization at 37 °C in a dark humid chamber, the slides were extensively washed in 2 × SSC/0.05% Tween 20 for 10 min, 50% formamide/0.5 × SSC for 20 min, 2 × SSC/0.05% Tween 20 for 20 min at 42 °C, and Tris-NaCl-Tween 20 buffer (TNT) for 5 min at room temperature.

After pretreatment with TNT buffer containing 0.5% blocking solution (Merck) for 30 min at 37 °C, the slides were incubated with 4 µg/mL of anti-digoxigenin fluorescence Fab fragments (Merck) and 1/1000-diluted streptavidin conjugated with DyLight™ 549 fluorescent dye (Vector Laboratories, Burlingame, CA) in TBST for 1 h at 37 °C in a dark humid chamber. After three washes with TBST and counterstaining with 0.4 μg/mL Hoechst 33342 (Thermo Fisher Scientific) in TBST, the slides were mounted with Fluoro-Keeper Antifade Reagent (Nacalai Tesque, Kyoto, Japan). Immunofluorescence images and DNA FISH images were obtained by a Microscope Axio Imager.A2 (Carl Zeiss, Jena, Germany) with a CCD camera (Carl Zeiss) and the software program AxioVision (Carl Zeiss).

### Slot-blot hybridization

Slot-blots were prepared as described^13^. In addition to the serial dilution series of the recombinant plasmid DNAs (eight dilution series of 0.1–300 ng DNA) and the insert PCR products containing each repetitive sequence used as a copy controlled standard, the germline and somatic genome DNA (eight dilution series of 1–3000 ng DNA) was transferred on membrane filters by a Bio-Dot SF slot blot apparatus (Bio-Rad, Hercules, CA).

Nine probes to detect EEEb1, EEEb2, EEEo1, EEEo2, EEPa1, EEPs1, EEEb4, EEEb5 and EEEb6 were newly synthesized, whereas the EEEb3 probe was prepared by an insert PCR using cloned plasmid DNA (Table 1). All probes were labeled with digoxigenin-11-ddUTP using the DIG Oligonucleotide 3′-End Labeling Kit, 2nd Generation (Merck) and then hybridized onto a membrane that had been denatured and renatured with 0.4 N NaOH and Tris–HCl buffer (pH 7.6). The hybridization and washing conditions were as described by the DIG system (Merck) according to the protocol recommended by the supplier. Chemiluminescent signals were detected with anti-digoxigenin-AP, Fab fragments and the chemiluminescent substrate CSPD™ ready-to-use (Merck). The detection and quantification of the chemiluminescent signals were performed as described by Nabeyama et al.^30^.

## Results

### Germline-specific amplification of rDNA-related sequences and the sequence analysis

**Figure 1:**
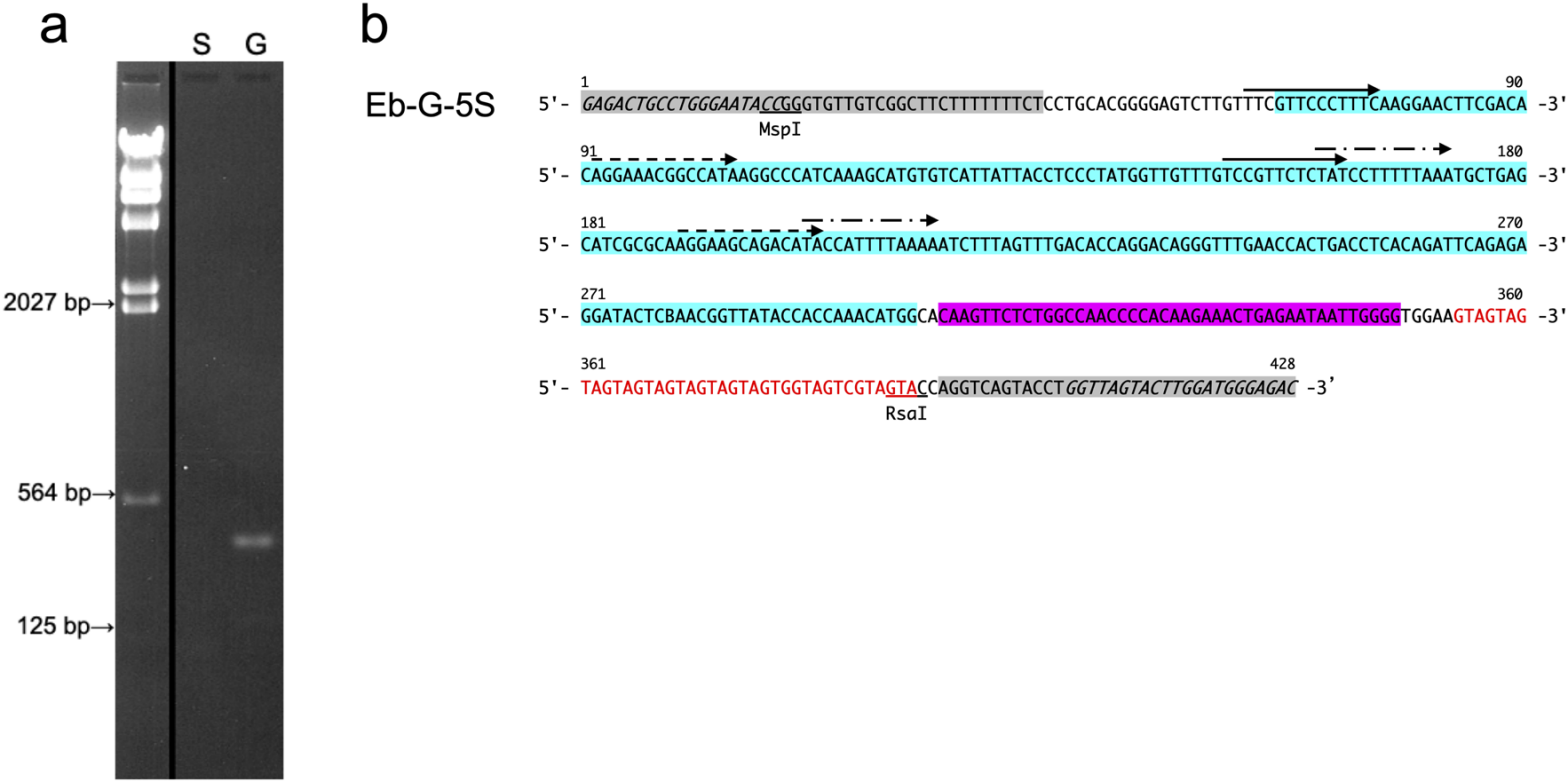
Amplification and consensus sequences of rDNA-related sequences. (**a**) PCR products using somatic DNA (lane S) and germline DNA (lane G) of the Japanese hagfish *E.* *burgeri* as templates were separated on a 1% agarose gel. The left lane in the photograph contains the DNA molecular size marker (Hind III-digested λ phage DNA). (**b**) The consensus nucleotide sequence was deduced from the inserts of germline genomic DNA clones. Gray shading: 5S rDNA, light-blue: SINE2-5_EBu, and purple: SINE2-6_EBu homologous regions. The GTA repeats detected are shown in red letters. Primer regions are indicated in italics. The direct repeats are marked with arrows. Recognition sites of each restriction endonuclease are underlined. The sequence data obtained here have been deposited in GenBank (LC667641).

We first tried to confirm whether 5S rDNAs were differentiated into the germline and somatic types as they are in some fish and whether the 5S rDNAs are eliminated from germline cells in the Japanese hagfish *E.* *burgeri*. PCR products from the germline and somatic genomes amplified with the primer designed based on the conserved region of 5S rDNA were separated on agarose gels, identifying a 380-bp DNA fragment that was exclusively detected from germline DNA (Fig. 1a); the PCR products were subsequently cloned into a TA-cloning vector. The Sanger sequencing of 14 positive clones demonstrated that the inserted DNA was composed of monomers or dimers of tandemly repetitive sequence, with a monomer length of 379–438 bp. The differences between each unit were shown to be single base substitutions and deletions in the 5S rDNA, a short interspersed nuclear element (SINE)2 family homologous region, and microsatellite-like GTA repeats. The consensus sequence was generated from a sequence alignment of 15-repeat sequences from 14 clones, designated as Eb-G-5S (Fig. 1b).

The GC content of the consensus sequence was 46.0%, and the intraspecific homology between the consensus and other sequences was 91.9–95.8% with an average identity of 92.7%. Three pairs of direct repeats (12, 13, and 14 bp) and triplet repeats (GTA) with two A to G/C substitutions were identified in the Eb-G-5S sequence. No continuous open reading frame (ORF) was detected on either strand. The homology search of the consensus sequence with BLAST revealed the partial homology to several sequences localized upstream or downstream of *Hox* genes (GenBank accession nos. MF182104–MF182109, MF398215–MF398219, MF398222, MF398223, MF398225, MF398227, MF398228, MF398231–MF398233, and MF398235)^34^ and *ParaHox* genes in *E.* *burgeri* (EU122194)^35^, plus SINE2-5_EBu and SINE2-6_EBu, a family of SINEs detected from *E.* *burgeri*^36^ and 5S rDNA in several animals (e.g. M10468)^37^. Unfortunately, all of the sequences showing partial homology have been located as a single copy but not tandemly repeated. These results demonstrated that Eb-G-5S consists of a fragment of 5S rDNA, a SINE2 family homologous region, and microsatellite-like GTA repeats (Fig. 1b).

### Identification of the sequences that are preferentially transcribed and eliminated from testis by the SSH technique

**Figure 2:**
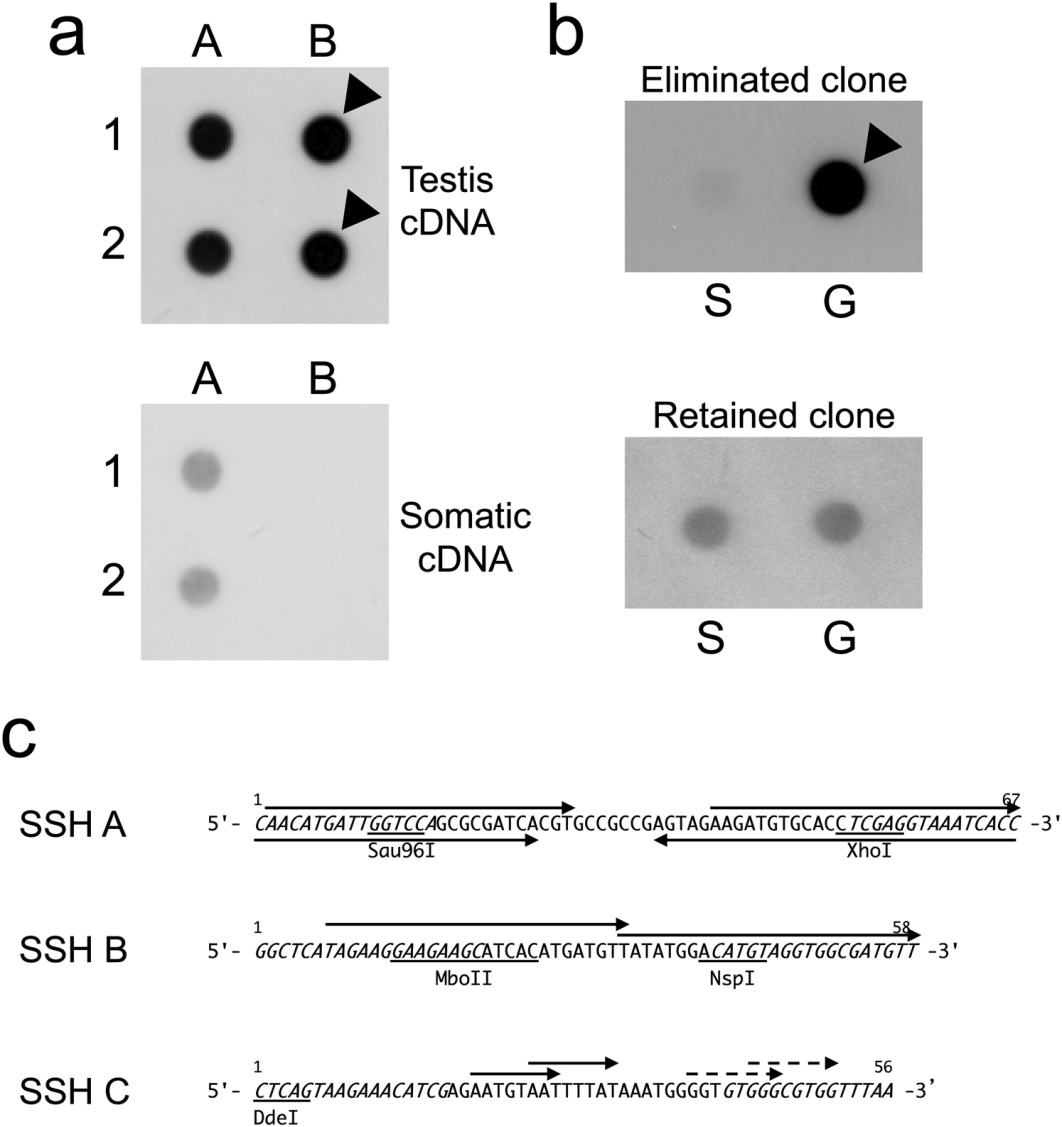
Representative dot-blots from the screening of cDNA clone and consensus sequences of three germline-specific repetitive DNA families. (**a**) Two identical membranes were dot-blotted with four cDNA clones and hybridized with probes prepared from *Rsa* I-digested testis cDNA (top) and *Rsa* I-digested somatic cDNA (bottom). The differentially expressed clones are indicated by arrowheads. (**b**) Membranes were blotted with somatic (S) and germline (G) genomic DNA and hybridized with probes prepared from each clone. The testis genome-specific signal is indicated by an arrowhead. (**c**) Each consensus nucleotide sequence was deduced from the inserts of germline genomic DNA clones. Upper arrows: the direct repeats. Lower arrows: the inverted repeats. The other notations correspond to those in Fig. 1. The sequence data obtained here have been deposited in GenBank (LC667642–LC667644).

We next used suppressive subtractive hybridization (SSH) to identify differentially expressed genes between the mature testis and somatic tissues (liver and blood) of the adult hagfish. The subtracted testis-specific cDNAs were cloned into the vector pCR2.1-TOPO, and then the positive clones were screened by two rounds of dot-blot hybridization. In 36 of 159 clones blotted on the membrane, the signal derived from the testis cDNA probe was clearly stronger than the signals from the somatic cDNA probe in the first screening, suggesting that these clones were specifically transcribed in the testis (Fig. 2a).

With the subsequent screening in which the plasmid DNA from 36 positive clones was separately hybridized with germline or somatic DNA blotted onto the membrane, a total of 23 clones were identified as an eliminated sequence according to specific hybridization signals on the germline DNA (Fig. 2b). The sequence analysis using 22 of 23 positive clones revealed that inserts of the clones were categorized into three tandemly repetitive DNA families, designated SSH A, B, and C, respectively.

The three repetitive DNA families newly amplified by PCR using germline genomic DNA with each primer (summarized in Table 1) were again re-cloned into plasmid vectors and sequenced. The consensus sequences of SSH A, B, and C were independently deduced from 15, 14, and 43 sequences examined, which revealed that the repeat units were 67-bp, 58-bp, and 56-bp, respectively (Fig. 2c). The GC content of the consensus sequence of the three repetitive families were 55.2%, 43.1%, and 37.5%, whereas the intraspecific nucleotide divergences among the sequences examined were 7.5%, 11.5%, and 8.8%, respectively. No continuous ORF was detected on either strand in all three families.

With the structural analysis, one pair of direct and inverted repeats, one pair of direct repeats, and two pairs of direct repeats were detected in SSH A, B, and C, respectively (Fig. 2c, arrows). The consensus sequences did not show any significant homologies by the homology search with BLAST.

### Southern-blot hybridization analysis of the repetitive DNA families

**Figure 3:**
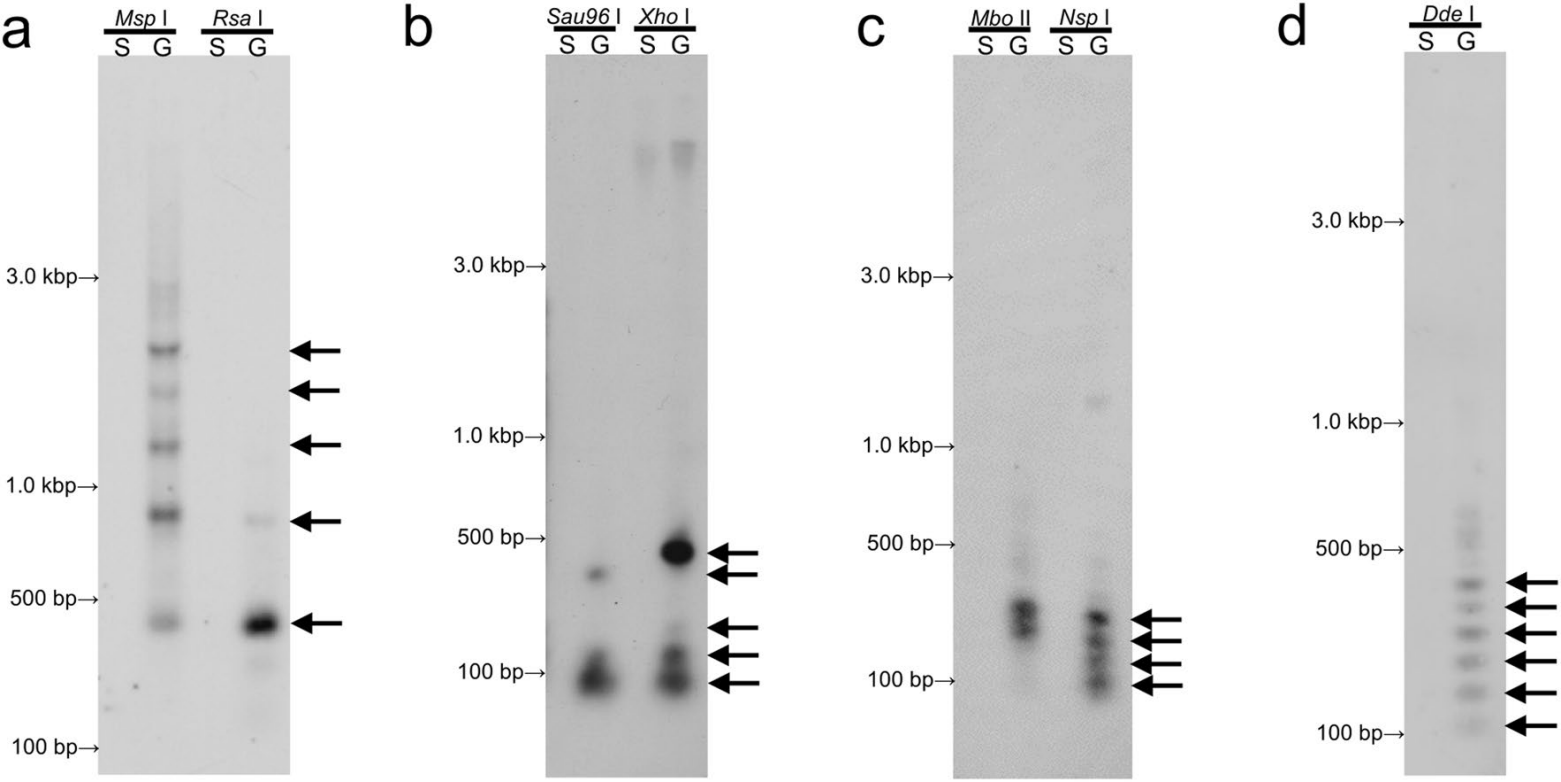
Southern-blot hybridization patterns of four DNA families. Germline DNA (lane G) and somatic DNA (lane S) digested with restriction enzyme *Msp* I, *Rsa* I, *Sau*96 I, *Xho* I, *Mbo* II, *Nsp* I or *Dde* I of *E.* *burgeri* were hybridized with digoxigenin-labeled Eb-G-5S (**a**), SSH A (**b**), SSH B (**c**), and SSH C (**d**) probes. Arrows indicate the corresponding size of the monomer and multimers of each DNA family.

We next compared the amount and genomic organization of the four novel repetitive sequences between the germline and somatic genomes of *E.* *burgeri* by Southern-blot hybridization. As shown in Fig. 3a, the signals of Eb-G-5S were all detected as ladder-like patterns digested with *Msp* I or *Rsa* I in the germline DNA but not in the somatic DNA, revealing that Eb-G-5S was tandemly repeated in the germline genome. The monomer of the repeat corresponding to approx. 380 bp was enriched in the *Rsa* I digest, and multimers (dimer to tetramer) were considerably enriched in the *Msp* I digest (Fig. 3a). These results suggested that the sequences recognized by *Rsa* I of the repeat units were highly conserved in the entire genome.

In SSH A, the signals were observed at the size corresponding to the monomer and their multimers in *Sau*96 I- and *Xho* I-digested germline DNA but not in somatic DNA (Fig. 3b). Interestingly, the major signal in the *Xho* I-digested germline DNA was detected as a 470-bp fragment equivalent to the heptamer, implying that this heptamer was the actual repeat unit of SSH A. In the case of SSH B, the tetrameric and pentameric bands were observed in the *Mbo* II-digested germline DNA. Additional bands corresponding to the trimer and dimer of SSH B were also observed in the *Nsp* I-digested germline DNA (Fig. 3c). In contrast, no signals of SSH B were ever detected in somatic DNA.

The multimeric ladder bands of SSH C were easily detected in the *Dde* I-digested germline DNA, although the monomer band was not detected (Fig. 3d). No signals of SSH C were observed in somatic DNA. All of the novel repetitive DNA families were tandemly repeated and specifically detected in the germline DNA but not in the somatic cells, and we thus designated the Eb-G-5S, SSH A, SSH B, and SSH C sequences as EEEb3, EEEb4, EEEb5, and EEEb6, respectively.

### Chromosomal mapping of the eliminated DNA families

**Figure 4:**
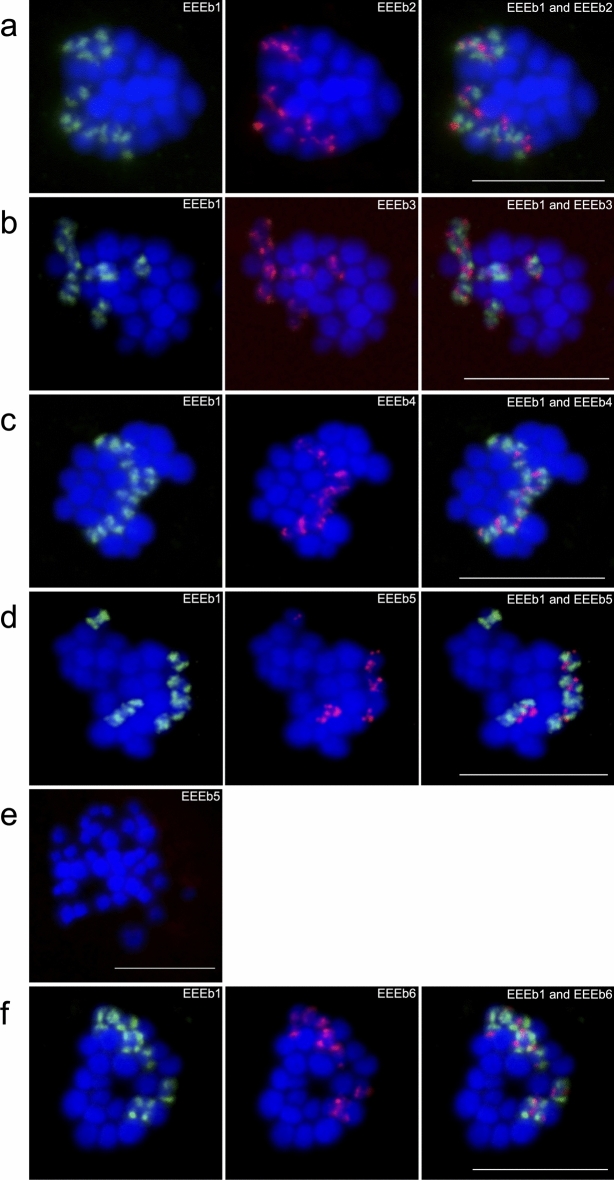
Chromosomal mapping of five eliminated DNA families in *E.* *burgeri*. Metaphase chromosomes from spermatocytes were hybridized using a digoxigenin-labeled EEEb1 probe (green) with biotin-labeled EEEb2 (**a**), EEEb3 (**b**), EEEb4 (**c**), EEEb5 (**d**), and EEEb6 (**f**) (red). The chromosomal localization of EEEb5 in somatic cells is shown in (**e**). Chromosomes were counterstained with Hoechst 33342 (blue). Scale bar = 5 μm.

The chromosomal localization of the eliminated DNA families EEEb2, EEEb3, EEEb4, EEEb5, and EEEb6 were thoroughly examined by multicolor FISH analyses with EEEb1, which was exclusively localized on all E-chromosomes. In EEEb2, fluorescent signals were clear on all EEEb1-positive but not EEEb1-negative chromosomes in the metaphases of the *E.* *burgeri* spermatocytes (Fig. 4a). The results of the colocalization analysis revealed that the signals of EEEb2 were mostly included in those of EEEb1, although EEEb2 was partly detected in the vicinity of EEEb1 signals. EEEb3 also appeared to be clustered on all EEEb1-positive chromosomes in the first meiotic metaphase, and EEEb1-negative chromosomes had no EEEb3 signals (Fig. 4b). The signals of EEEb3 rarely overlapped with those of EEEb1.

In the case of EEEb4, the major signals seemed to be colocalized with those of EEEb1, and several minor signals were observed at the regions adjacent to the EEEb1 signals on the EEEb1-positive chromosomes in metaphasic spermatocytes. No signals of EEEb4 were detected on EEEb1-negative chromosomes (Fig. 4c). The signals of EEEb5 were detected on all EEEb1-positive chromosomes in the spermatocytes. The intense signals seemed to be located adjacent to EEEb1 signals on the chromosomes (Fig. 4d). On the other hand, no signals of EEEb5 were observed on EEEb1-negative chromosomes (Fig. 4e).

The EEEb6 signals were detected on all EEEb1-positive chromosomes in spermatocytes as dense signals (Fig. 4f). The distribution of EEEb6 signals frequently matched with those of EEEb1. The signals were absent from EEEb1-negative chromosomes. The same results were obtained from mitotic spermatogonia metaphases (Supplementary Fig. S1).

**Figure 5:**
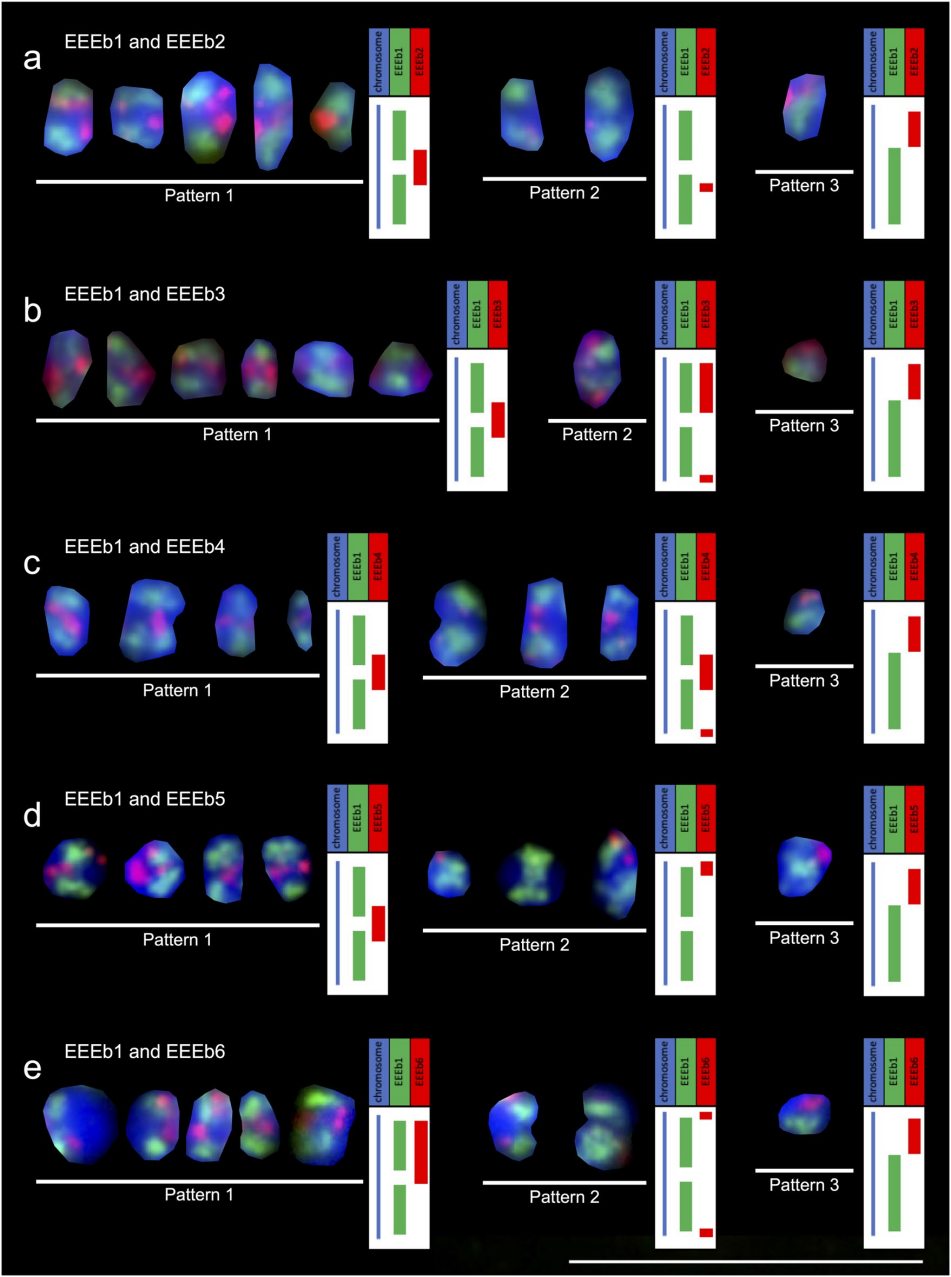
Karyogram of E-chromosomes and schematic diagrams of six eliminated DNA families in *E.* *burgeri*. Eight pairs of E-chromosomes were divided into three patterns (Pattern 1 to 3) by their signal localizations. Schematic diagrams were shown at the right of chromosomal images of each signal distribution patterns. Scale bar = 5 μm. The other notions correspond to those to in Fig. 4.

Although these signal localizations were quite similar, the signal distributions were divided into three patterns in each combination as summarized in Fig. 5. In EEEb1, the signal localization patterns were divided into two patterns; (*i*) two cluster were symmetrically located on the terminal regions of seven pairs of E-chromosomes (Fig. 5, Pattern 1 and Pattern 2), and (*ii*) signal cluster was located on the terminal region of one pair of E-chromosomes (Fig. 5, Pattern 3). On the other hand, the distribution of the other DNA families (EEEb2 to 6) were divided into three patterns; (*i*) the signals were located on the middle regions of E-chromosomes with symmetric EEEb1 clusters (Pattern 1), (*ii*) the signals were located on the interstitial and/or terminal regions of E-chromosomes with symmetric EEEb1 clusters (Pattern 2), and (*iii*) the signals were located on the terminal region of E-chromosomes with unsymmetric EEEb1 cluster (Pattern 3).

All five repetitive DNA families were selectively located on all E-chromosomes, and this was corroborated not only by the elimination of novel repetitive DNA families confirmed by our Southern-blot analysis but also by the elimination of EEEb2 observed by Kubota et al.^11^.

### Slot-blot hybridization of the eliminated DNA families

To quantify the amounts of the 10 eliminated DNA families (EEEb1–6, EEEo1, EEEo2, EEPa1, and EEPs1) in the somatic and germline genomes of *E.* *burgeri*, we performed slot-blot hybridization with each repetitive sequence-specific DNA probe. The signal intensities in the germline and somatic genomic DNAs were standardized with the copy-controlled standards, and the copy number and total amount of each repetitive DNA family were calculated (summarized in Table 2). EEEb1, which appeared to be entirely distributed in all E-chromosomes, was most abundant. The copy number and DNA amount in the diploid germline genome were approx. 5.5 × 10^6^ and 5.4%, respectively.

**Table 2:** Summary of the eliminated elements in *E. burgeri.*

Eliminated element	Unit size of repeat, bp	G + C content, %	Nos. of copies of diploid genome (percentage of diploid genome)		Proportion of the eliminate copy no., %
			Somatic cell	Germ cell	
EEEb1	64	59.4	3.8 × 10^2^ [4.8 × 10^−4^%]	5.5 × 10^6^ [5.4%]	> 99%
EEEb2	57	26.3	− [−]	3.9 × 10^5^ [3.4 × 10^−1^%]	> 99%
EEEb3	404–446	45.1	6.7 × 10^4^ [5.6 × 10^−1^%]	9.8 × 10^4^ [6.4 × 10^−1^%]	31.6%
EEEb4	67	55.2	6.4 × 10^4^ [8.0 × 10^−2^%]	9.7 × 10^5^ [1.0%]	93.4%
EEEb5	58	43.1	6.2 × 10^2^ [7.1 × 10^−4^%]	8.9 × 10^4^ [8.0 × 10^−2^%]	> 99%
EEEb6	56	37.5	6.3 × 10 [6.9 × 10^−5^%]	1.0 × 10^6^ [8.8 × 10^−1^%]	> 99%
EEEo1	161	40.4	4.5 × 10^4^ [1.4 × 10^−1^%]	9.4 × 10^4^ [2.2 × 10^−1^%]	52.1%
EEEo2	84	29.8	1.3 × 10^4^ [1.7 × 10^−2^%]	1.7 × 10^4^ [2.2 × 10^−2^%]	23.5%
EEPa1	83	38.6	7.0 × 10^4^ [1.9 × 10^−1^%]	3.6 × 10^5^ [4.6 × 10^−1^%]	80.6%
EEPs1	39–40	60	5.9 × 10^2^ [3.7 × 10^−4^%]	7.8 × 10^2^ [4.8 × 10^−4^%]	24.4%

EEPs1 showed the lowest copy number and amount in the germline genome (7.8 × 10^2^/diploid, 4.8 × 10^−4^%). Our results for EEEb1 are threefold lower than those reported by Kubota et al.^11^. In total, the 10 eliminated DNA families accounted for approx. 9.0% of the total germline genomic DNA and 43.3% of the total eliminated DNA.

In addition, our quantification of hybridization signals revealed that (*i*) almost all of the repetitive DNA families were partially retained in the somatic genome, and (*ii*) somatically retained repetitive sequences accounted for < 1.0% of the somatic genomic DNA. Although most of the copies (> 80%) were discarded in EEEb1, EEEb2, EEEb4, EEEb5, EEEb6, and EEPa1, we observed that 68.4%, 47.9%, 76.5%, and 75.6% of the copy numbers of EEEb3, EEEo1, EEEo2, and EEPs1, respectively, were maintained in somatic cells. Therefore, even though not all copies of these repetitive sequences are lost in somatic cells, all of these repetitive sequences appear to have been amplified selectively in the germline genome and were eliminated.

## Discussion

This investigation identified four novel repetitive DNA families as the eliminated sequences of the Japanese hagfish *E.* *burgeri*. All families named EEEb3 to EEEb6 were tandemly repeated and ubiquitously present on all E-chromosomes, amounting to approx. 2.6% of their germline genome. EEEb2 (which was previously identified as an eliminated family in this species) also showed localization similar to that of EEEb1 and EEEb3–6. According to the calculation of copy numbers by slot-blotting, the 10 eliminated DNA families accounted for 9.0% of the germline diploid genome, whereas the ratio of the eliminated copies from the somatic genome varied from almost 100% to 23.5% for each family. These results demonstrate that the ten DNA families were selectively amplified in the germline genome of *E.* *burgeri*.

In our investigation to determine whether truncated 5S rDNA were discarded during chromosome elimination, the tandem repeat family, EEEb3 consisting of a SINE2 retrotransposon-like sequence (showing > 80% homology), GTA repeats, and a partial sequence of 5S rDNA was exclusively isolated from the germline genome (Fig. 1b). A similar situation was also observed in sea lamprey. One of eliminated sequences in sea lamprey, *Germ1*, is composed of a somatically rare (SR) region and a truncated 28S rDNA sequence. Since the boundary between this SR region and the 28S rDNA fragment is identical to the 3′ integrated sequence after the transposition of *R2* retrotransposon, the SR region is a sequence derived from the *R2* retrotransposon^17^. The SR region of *Germ1* has been consistently mapped to major rDNA loci on eight meiotic bivalent chromosomes in germ (testis) cells, whereas in the somatic cells the SR region and 28S rDNA are restricted to a single mitotic chromosome pair^14,38^.

Other studies suggested that the integration between distinct retrotransposons had occurred frequently in an ancestor of *E.* *burgeri* by a chimeric formation of SINE1 and SINE2 families, possibly generated by DNA recombination and/or one or more switching template RNAs mechanisms^36,39^. Those findings suggested that one of the biological functions of chromosome elimination (or chromatin diminution) in hagfish and sea lamprey may be the exclusion of these dysfunctional rDNA copies. In the present study, the slot-blot assay revealed that approx. two-thirds of EEEb3 copies present in the germline genome are retained in the somatic genome (Table 2), whereas EEEb3 was not detected on the somatic genome by the PCR, Southern-blotting, or FISH analyses (Figs. 1a, 3a, 4b). This result may be explained by structural variations of EEEb3 between the germline and somatic genomes. All of the EEEb3 clones isolated from the somatic genome lacked the 5S rDNA region and GTA repeats, with no exception, suggesting that selective elimination of tandemly repeated EEEb3 harboring 5S rDNA, SINE2, and GTA repeats (Supplementary Fig. S2). It was thus difficult to detect the truncated EEEb3 in the somatic genome by Southern-blotting, and the signals derived from truncated EEEb3 and SINE2 repeats were overestimated in the germline/somatic genome.

In adult *E burgeri*, all of the E-chromosomes but not the remaining non-E-chromosomes are universally maintained as a heterochromatic chromosome in the mitotic and meiotic metaphase in germ cells^7^, but the mechanism underlying the heterochromatin formation is still unknown. It has been reported that small non-coding RNAs (ncRNAs) are transcribed from repetitive sequences in constitutive heterochromatin regions such as the centromere in several animals^40–46^. In fission yeast, transcribed ncRNAs play a crucial role in the establishment of the heterochromatin at the centromere through cis-acting RNA interference (RNAi)^40,47^. Like in fission yeast, repetitive DNA-associated RNAi machineries in other animals also induce heterochromatinization of other regions^41–43,46^.

In the present study, EEEb4, EEEb5, and EEEb6 were isolated as eliminated elements that were transcribed specifically in the *E.* *burgeri* testis, but there is as yet no evidence regarding whether all copies in the germline genome were actually and equally transcribed. Since the slot-blot analysis revealed that > 90% of these families were excluded from the somatic genome during chromosome elimination (Table 2), we attributed the transcriptional silencing of these families in the somatic cells to the loss of those copies in the somatic genome. Taking all of these results into account, we propose that it is highly likely that EEEb4, EEEb5, and EEEb6 are also involved in RNAi-mediated heterochromatinization in the heterochromatin formation of this hagfish species. On the other hand, in molecular biology approaches, especially SSH experiment, the protein coding genes present on the E-chromosomes have never identified. This may be caused by the limitation of detection sensitivity of SSH technique, perhaps, due to low sequence frequency of the protein coding genes in testis cDNA library. Hence, we are now conducting the next generation sequencing analysis using hagfish germline and somatic genomes to find the eliminated protein coding genes with low frequency in testis cDNA library.

All of the eliminated DNA families, including the sequences isolated here and the previously reported sequences, can be divided into several categories based on their sequence characteristics (Table 2). The first group consists of 56- to 84-bp repeats harboring sub-repeats (small direct repeats) within the repeating unit, and the members of this group are distributed tandemly in the germline genome. Six of the 10 eliminated DNA families (EEEb2, EEEb4, EEEb5, EEEb6, EEEo2 and EEPa1) belonged to this group, and all except EEEb4 are AT-rich (EEEb4 is GC-rich). These families account for approx. 2.2 × 10^−2^% to 1.0% of the germline genome, and EEEb2, EEEb4, EEEb5, and EEEb6 are almost completely absent in the somatic genome. EEEb6 and EEPa1 were observed to contain several pairs of short sub-repeats which covered approximately half of the region of their repeating units (Kubota et al.^10^; Fig. 2c). EEEb2 and EEEo2 are divided into three homologous sub-repeats, whereas EEEb4 and EEEb5 each have two homologous sub-repeats (Nabeyama et al.^30^; Kubota et al.^9,11^; Fig. 2c). As suggested in our previous studies, these DNA families might have evolved by saltatory replication events^9,30^.

The second group of eliminated DNA families (EEEb1 and EEPs1) are GC-rich sequences that had inverted repeats within the repeat unit and were highly tandemly aligned in the germline genome. The inverted repeats present in both families potentially form a hairpin structure in the single-stranded DNA and/or RNA^11,13^. EEEb1 was exclusively located on all of the E-chromosomes, and the amounts in the eliminated genome exceeded those of other repetitive DNA families (Kubota et al.^11^; Table 2). It can thus be speculated that the E-chromosomes of *E.* *burgeri* consist mostly of the EEEb1 family.

The last group of eliminated DNA families (EEEb3 and EEEo1) is composed of the interspersed DNA repeats that have moved around the genome and are occasionally arrayed in tandem. EEEb3 was tandemly arrayed in the E-chromosomes of germ cells, whereas truncated EEEb3 was dispersed in the somatic chromosome mentioned above. In the case of EEEo1, the sequences showing high homology to EEEo1 have been found in the vicinity of *Hox* genes (MF182102–MF182109, MF398215–MF398223, MF398225, and MF398227–MF398235)^34^, *ParaHox* genes (EU122194)^35^, and variable lymphocyte receptor genes -A and -B (*VLR-A* and *VLR-B*) in *E.* *burgeri* (AY965678 and AY965679)^48^.

In contrast to this dispersed distribution, the tandem arrangement of the EEEo1 family in the germline genome was also verified by PCR amplification^30^. EEEb3 appears to be generated through the 5S rDNA duplication, because it has part of the 5S rDNA sequence within the repeat unit (Fig. 1a). Although it is not yet known how to amplify these repetitive sequences in the germline genome, further investigations of the amplification mechanism of the multigene family can contribute to our understanding of the evolution of eliminated genomes in hagfish species.

**Figure 6:**
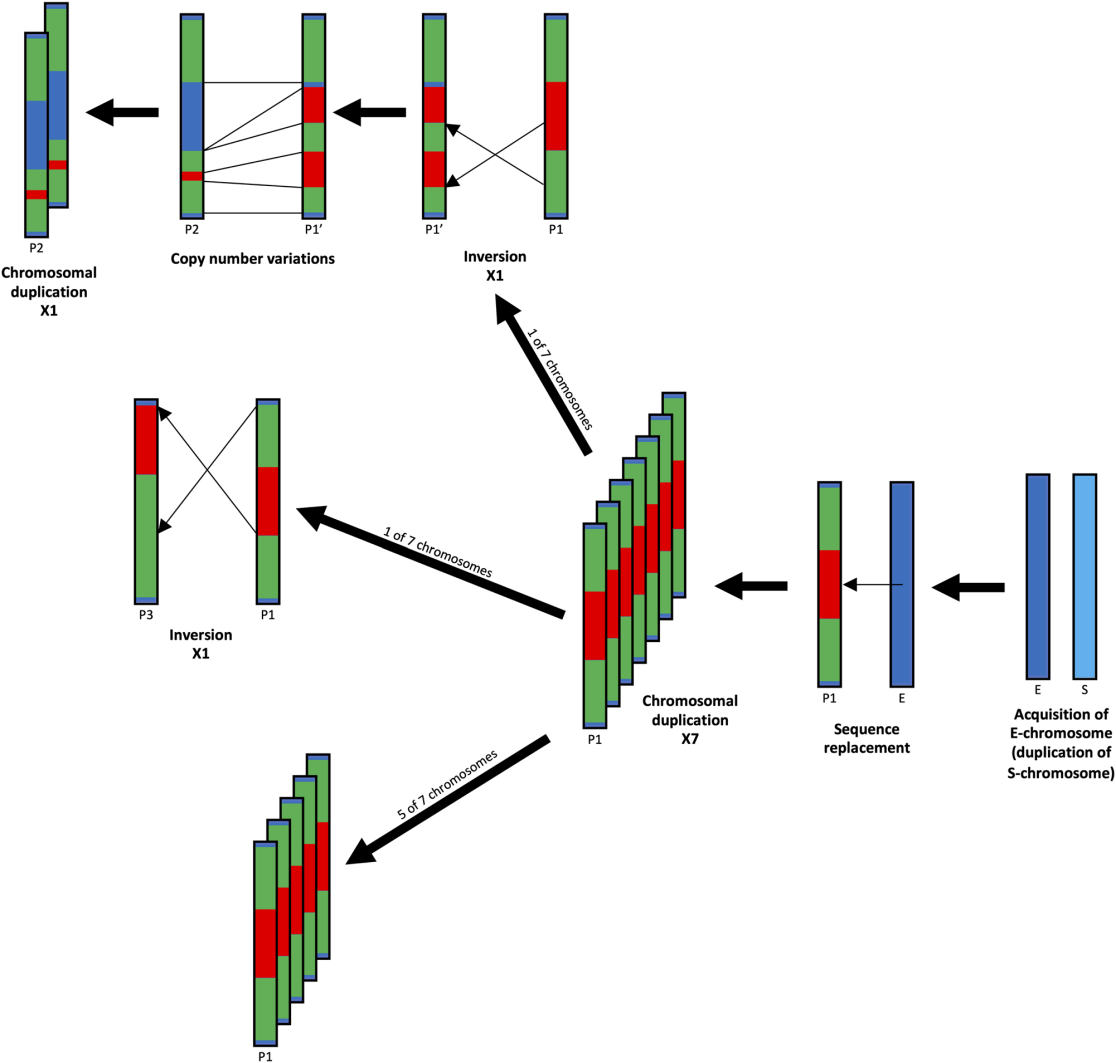
A hypothetical scheme of the evolution of E-chromosomes in *E.* *burgeri*. This representation is based on the results of FISH using EEEb1 to EEEb6 presented in this study (Fig. 5). Single P1 (Pattern 1) E-chromosomes containing EEEb1 (green) and other EEEb families (red) were duplicated seven times via meiotic drive after the comprehensive sequence replacement of E-chromosomes (E). P2 (Pattern 2) E-chromosomes were formed by a series of single chromosomal inversion, copy number variations and single chromosomal duplication of one of seven P1 E-chromosome. P3 (Pattern 3) E-chromosome were formed by single chromosomal inversion of one of P1 E-chromosome. Homogenization mechanisms retained the P1–3 E-chromosomes during a long evolutionary period. Before the sequence replacement, E-chromosome duplicated from somatically retained chromosome (S).

In animals undergoing chromosome elimination (chromatin diminution) including hagfish, E-chromosomes are mostly heterochromatic and composed of many repetitive DNA families^9–12,14,49–51^. Although many studies have attempted to elucidate the evolutionary process of chromosome elimination, the origin and the differentiation of E-chromosomes in any species remains unclear. The results of our present study using FISH analysis demonstrates that EEEb1 to EEEb6 were selectively detected on all E-chromosomes in the spermatocytes of *E.* *burgeri* with quite similar signal distributions (Figs. 4, 5). This suggests that that the eight pairs of E-chromosomes are derived from a single pair of ancestral chromosomes that underwent multiple duplication events caused by a meiotic drive over a long evolutionary period (Fig. 6; lower panel). In support of this hypothesis, in the genus *Eptatretus*, the number of E-chromosomes varies among examined species (2n = 14–62), and some of them have supernumerary E-chromosomes, B chromosomes (which are additional dispensable chromosomes that occur frequently among multicellular organisms)^8^. On the other hand, the signal distribution of repeated elements examined present study (EEEb1–6) is slightly different between E-chromosomes (Fig. 5), which could be driven by differences in repeat copy number and chromosomal rearrangements, such as chromosomal inversion (Fig. 6; upper and middle panels), between E-chromosomes over time. The origination of E-chromosomes was reported in chironomid and songbirds, in which germline-restricted chromosomes (GRCs) are also eliminated during early embryogenesis^52,53^. In these taxa, microdissected GRC-specific probes were clearly detected on not only GRC but also a pair of autosomal chromosomes to be retained in the somatic cells^54,55^.

In the case of B chromosomes, it is known that the B chromosomes also originate from fragmented or degenerated autosomal chromosome complement in phylogenetically divergent species^56–63^. It has been proposed that chromosome elimination was acquired in the ancestral species of Cyclostomata before the Petromyzontiformes-Myxiniformes divergence^4,15,16^, at least 400 million years ago^64^. Bachmann-Waldmann et al.^65^ hypothesized that chromosome elimination accelerated the nucleotide divergence of the eliminated sequences. In accordance with these findings, in *E.* *burgeri*, the E-chromosomes seem to have diverged rapidly over a period of 400 million years following their origination from somatic chromosomes, and have completely lost the ancestral sequences shared with the somatic chromosomes (unlike chironomid and songbirds). The original ancestral chromosome of 16 E-chromosomes thus appears to have degenerated or been replaced by other elements, since EEEb1 to EEEb6 were never detected on non-E-chromosomes (Fig. 6; right panels). In support of this hypothesis, the similar result was reported from one of the other hagfish species, *E. cirrhatus*; all of three *E. cirrhatus*-specific eliminated DNA families, EEEc1–3 were detected on their E-chromosomes but not on somatically retained chromosomes by FISH analysis^12^.

In contrast to the genomic novelty of the origin of the E-chromosomes, our FISH analysis using EEEb1 to EEEb6 clearly revealed the chromosomal conservation between E-chromosomes of *E. burgeri*, suggesting the existence of an autonomous (homogenization) mechanism, such as a recombination-mediated DNA repair system, to maintain the array of repetitive sequences in this species during the > 400 million-year period. In support of this hypothesis, the meiotic metaphase spreads tended to show clusters of dumbbell-shaped bivalent E-chromosomes in *E. burgeri*^7^.

The contradiction of genomic novelty and sequence homogenization within the E-chromosomes could be explained by birth-and-death and concerted evolution of repetitive DNA families^66^. The birth-and-death model assumes that new sequences are generated by repeated duplication, and some duplicated sequences remain in the genome for a long evolutionary period, whereas others are deleted or dysfunctionalized through deleterious mutations. On the other hand, in the concerted evolution model, repetitive sequences are assumed to evolve in a concerted manner with a mutation through the entire member by repeated unequal crossover leading to intraspecific sequence homogeneity. Further investigations of both repetitive sequences and protein-coding sequences will help us understand the evolution of E-chromosomes and chromosome elimination.

## Supplementary Information

Supplementary Information 1.

Supplementary Information 2.

## Data Availability

Sequence data have been deposited in GenBank (accession nos. LC667641–LC667644 and LC669413–LC669417).
