# Supplementary material for: Novel selectively amplified DNA sequences in the germline genome of the Japanese hagfish, Eptatretus burgeri
Source: Sci Rep. 2022 Dec 9;12:21373. doi: 10.1038/s41598-022-26007-2 (PMC9734144; doi:10.1038/s41598-022-26007-2)
Supplement: Supplementary file 1 — Supplementary Information 1. [file 41598_2022_26007_MOESM1_ESM.pdf]

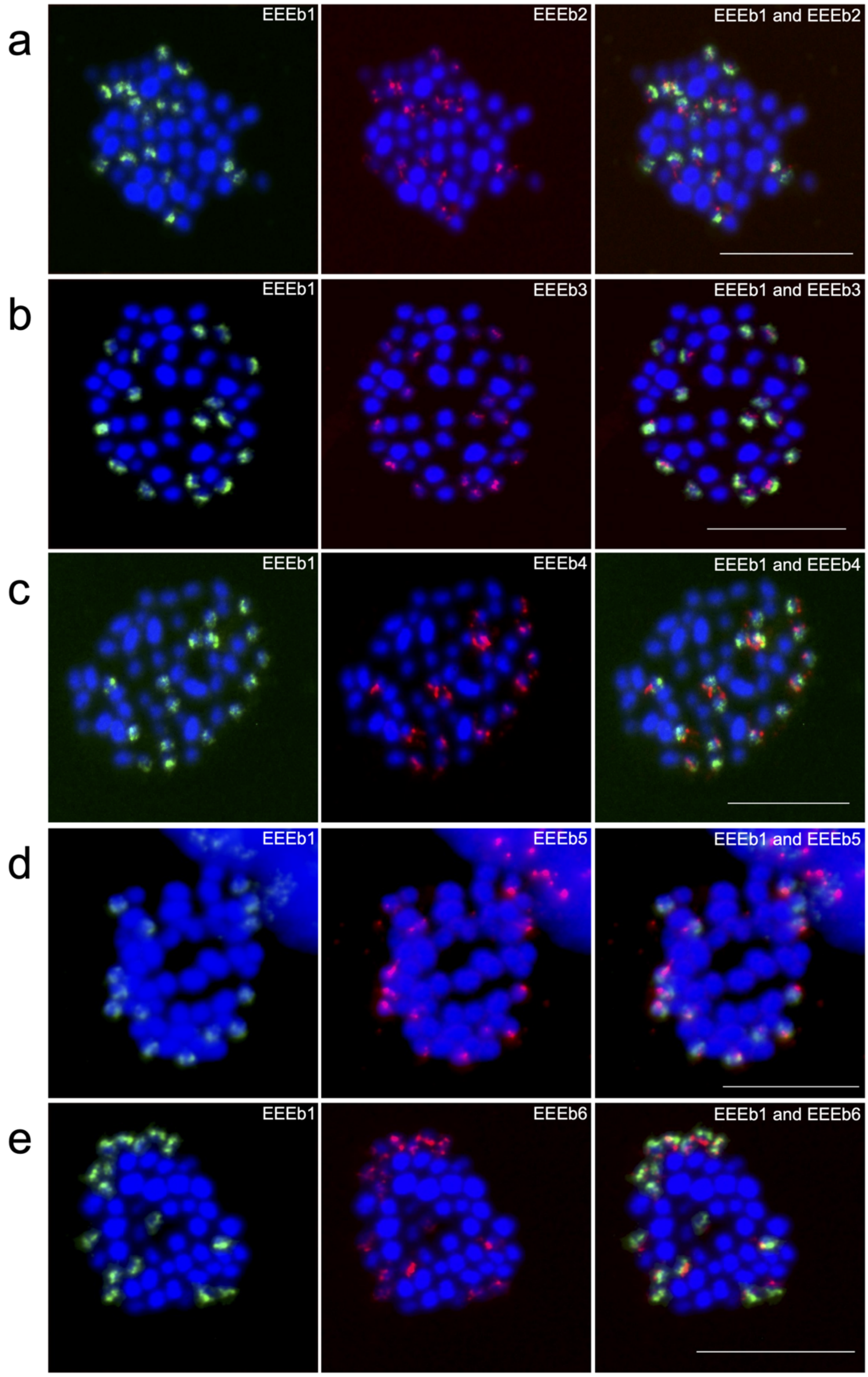

Supplementary Figure S1. Chromosomal mapping of five eliminated DNA families in the Japanese hagfish *E. burgeri*. Metaphase chromosomes in spermatogonia were

hybridized using a digoxigenin-labeled EEEb1 probe (*green*) with biotin-labeled EEEb2 (**a**), EEEb3 (**b**), EEEb4 (**c**), EEEb5 (**d**), and EEEb6 (**e**) (*red*). Chromosomes were counterstained with Hoechst 33342 (*blue*). Scale bar = 5 μm.

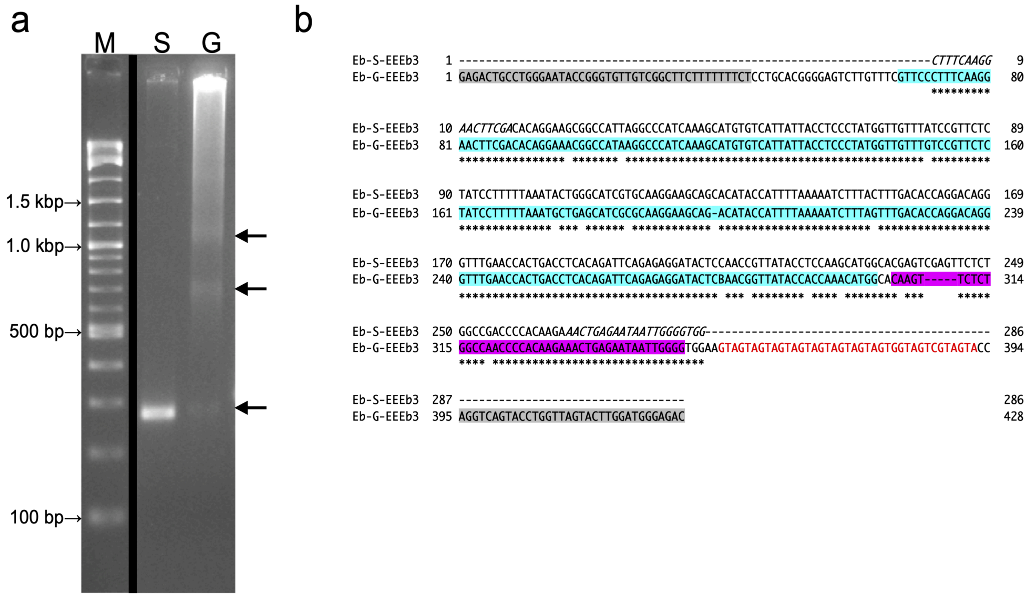

Supplementary Figure S2. Amplification and comparison of EEEb3 from the somatic genome and that from the germline genome. a: PCR products using somatic DNA (lane S) and germline DNA (lane G) of *E. burgeri* as templates were separated on a 3% agarose gel. The *left lane* in the photograph shows the DNA molecular size marker (2-Log DNA Ladder). **b:** The consensus sequence of EEEb3 from the somatic genome was deduced from three units from three clones, designated as Eb-S-EEEb3 (*top*), and aligned with EEEb3 from the germline genome (Eb-G-5S), here designated as Eb-G-EEEb3 (*bottom*). Dashes (–) represent alignment gaps. *Identical nucleotides between consensus sequences. Base substitutions are indicated by the respective bases. *Gray shading:* 5S rDNA, *light-blue:* SINE2-5_EBu, and *purple:* SINE2-6_EBu homologous regions. GTA repeats detected are in *red letters*. Primer regions are in *italics*. The sequence data obtained here have been deposited in GenBank (LC669414).
