# Supplementary material for: Novel selectively amplified DNA sequences in the germline genome of the Japanese hagfish, Eptatretus burgeri
Source: Sci Rep. 2022 Dec 9;12:21373. doi: 10.1038/s41598-022-26007-2 (PMC9734144; doi:10.1038/s41598-022-26007-2)

Figure 1a

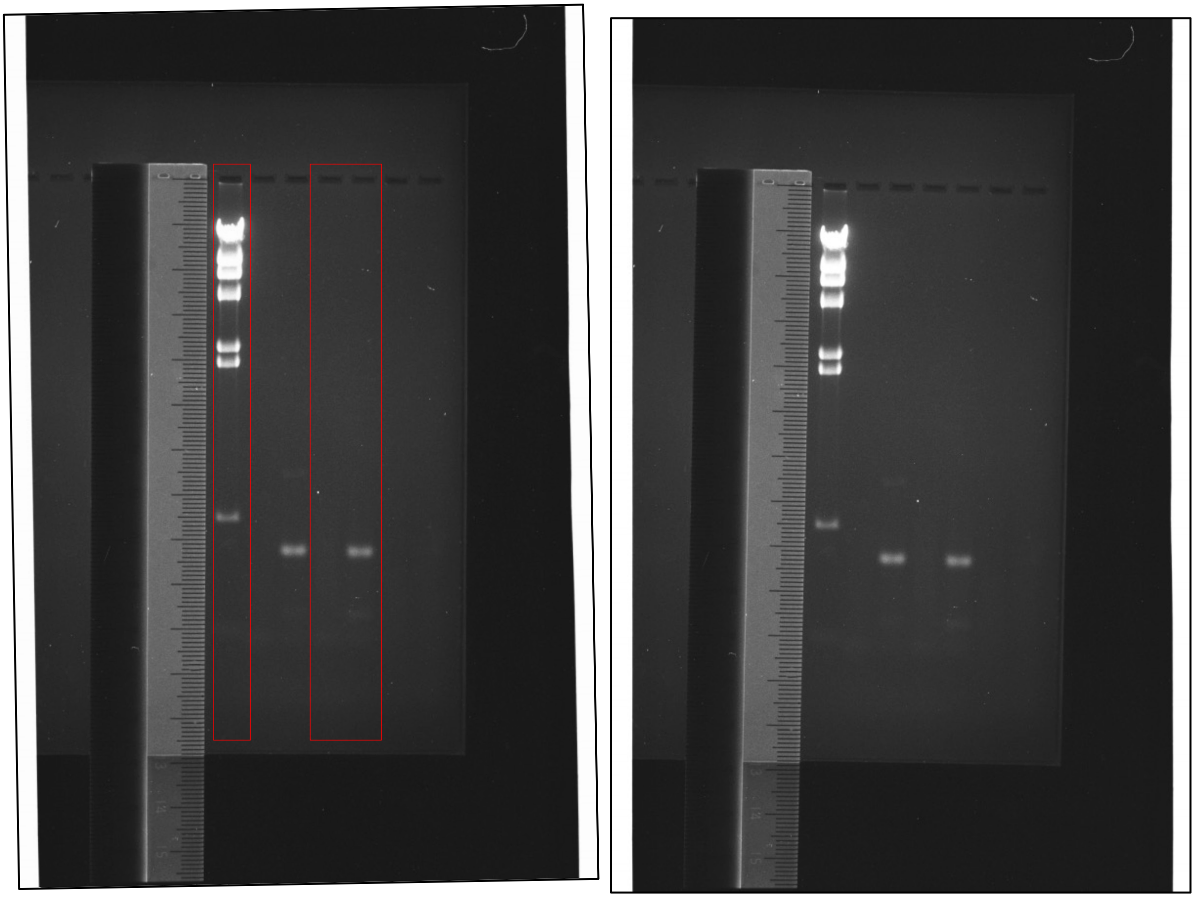

Figure 2a

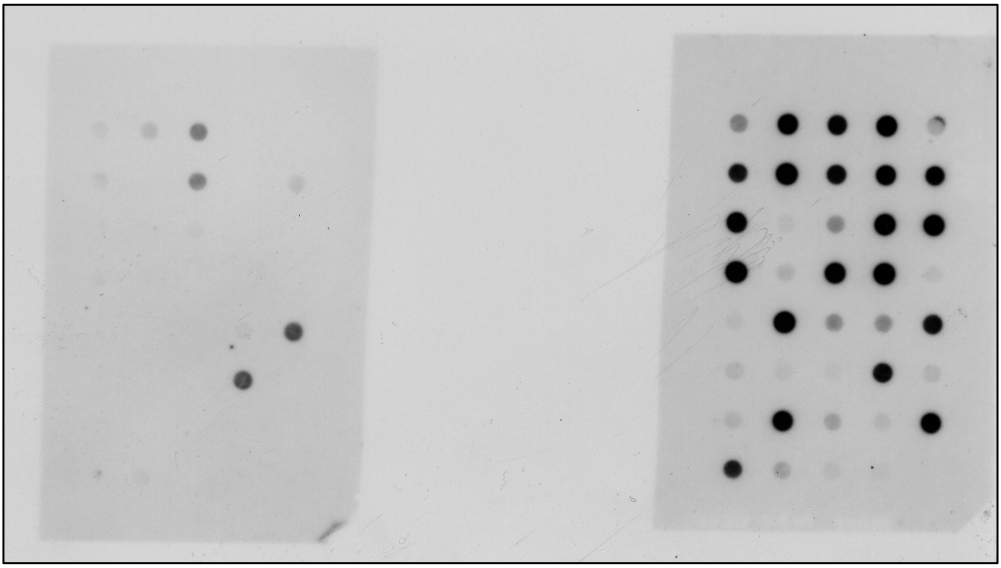

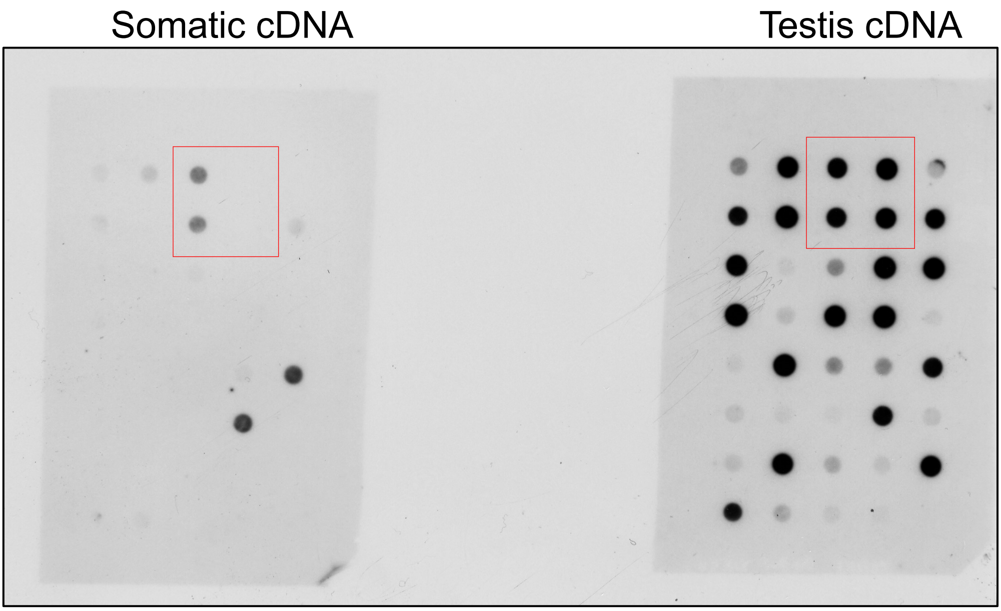

Figure 2b

Eliminated clone

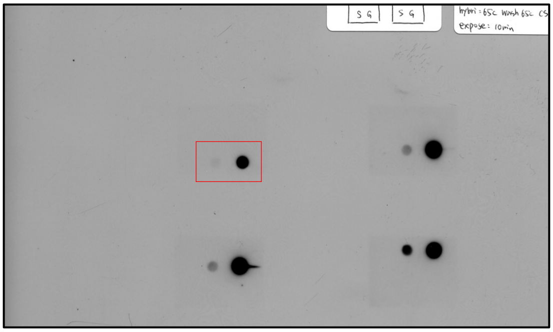

Retained clone

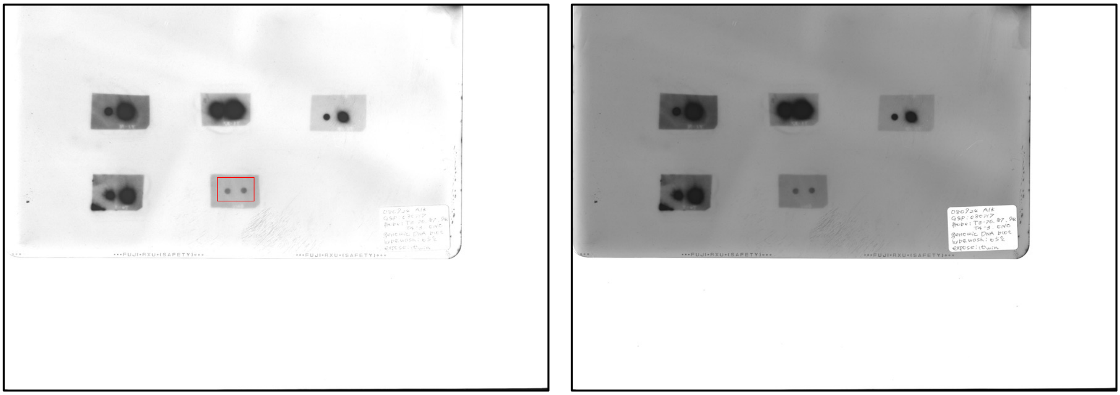

Figure 3a

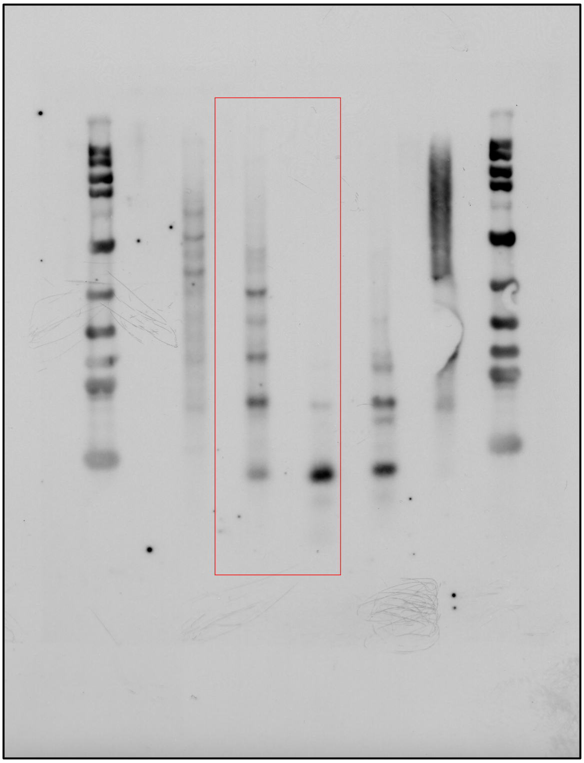

Figure 3b

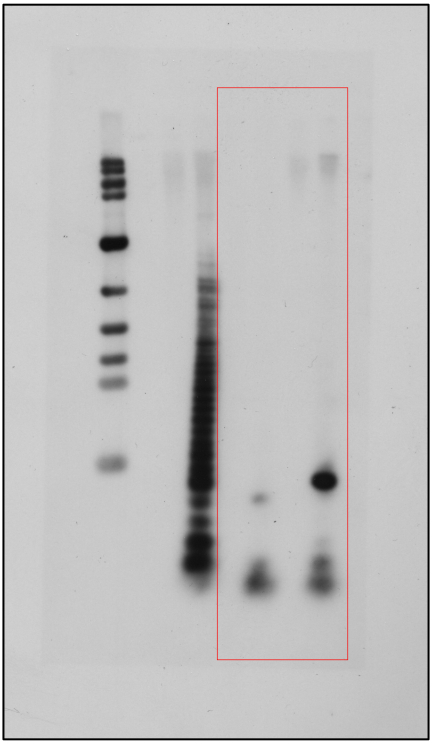

Figure 3c

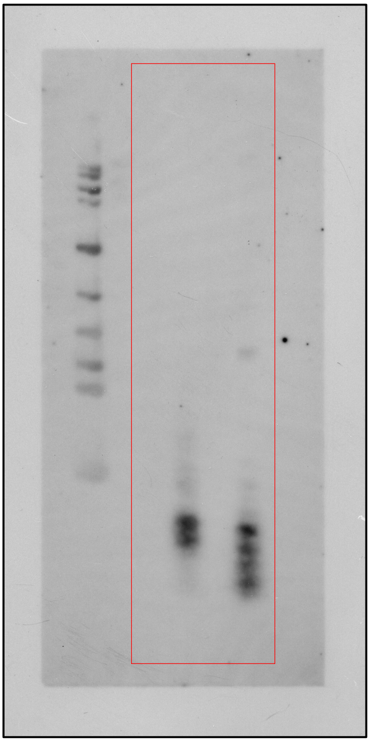

Figure 3d

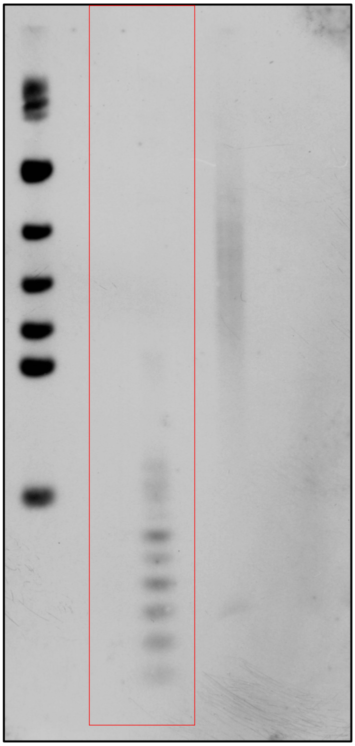

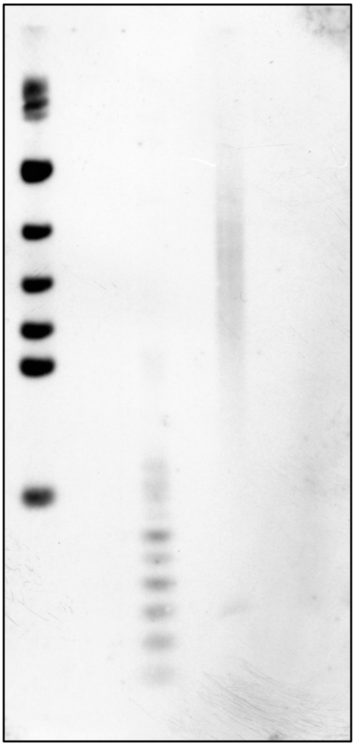

Supplementary Figure S2a

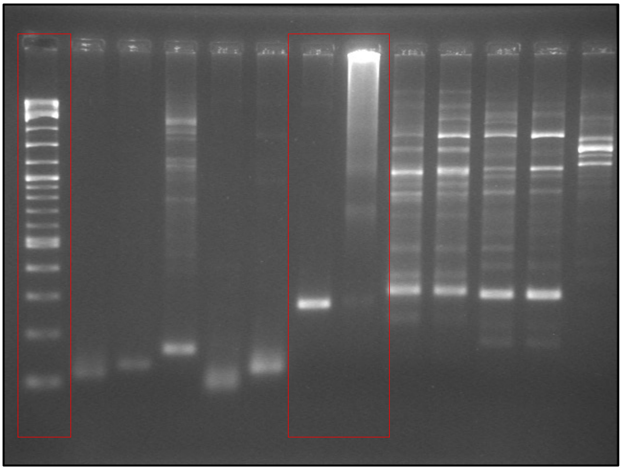

Supplement: Supplementary file 2 — Supplementary Information 2. [file 41598_2022_26007_MOESM2_ESM.pdf]
